# Supplementary material for: Ultra-depleted hydrogen isotopes in hydrated glass record Late Cretaceous glaciation in Antarctica
Source: Nat Commun. 2022 Sep 7;13:5209. doi: 10.1038/s41467-022-32736-9 (PMC9452555; doi:10.1038/s41467-022-32736-9)
Supplement: Supplementary file 2 — Description of Additional Supplementary Files [file 41467_2022_32736_MOESM2_ESM.pdf]

Description of Additional Supplementary Files

File name: Supplementary Data 1

Description: hydrogen isotope composition data (.xlsx)

File name: Supplementary Data 2

Description: d'17O data (.xlsx)

File name: Supplementary Data 3

Description: In-situ 40Ar/39Ar analytical locations (.pdf)

File name: Supplementary Data 4

Description: 40Ar/39Ar Geochronology Data (.xlsx)
