# Supplementary figures and images for: Ultra-depleted hydrogen isotopes in hydrated glass record Late Cretaceous glaciation in Antarctica
Source: Nat Commun. 2022 Sep 7;13:5209. doi: 10.1038/s41467-022-32736-9 (PMC9452555; doi:10.1038/s41467-022-32736-9)

BR10

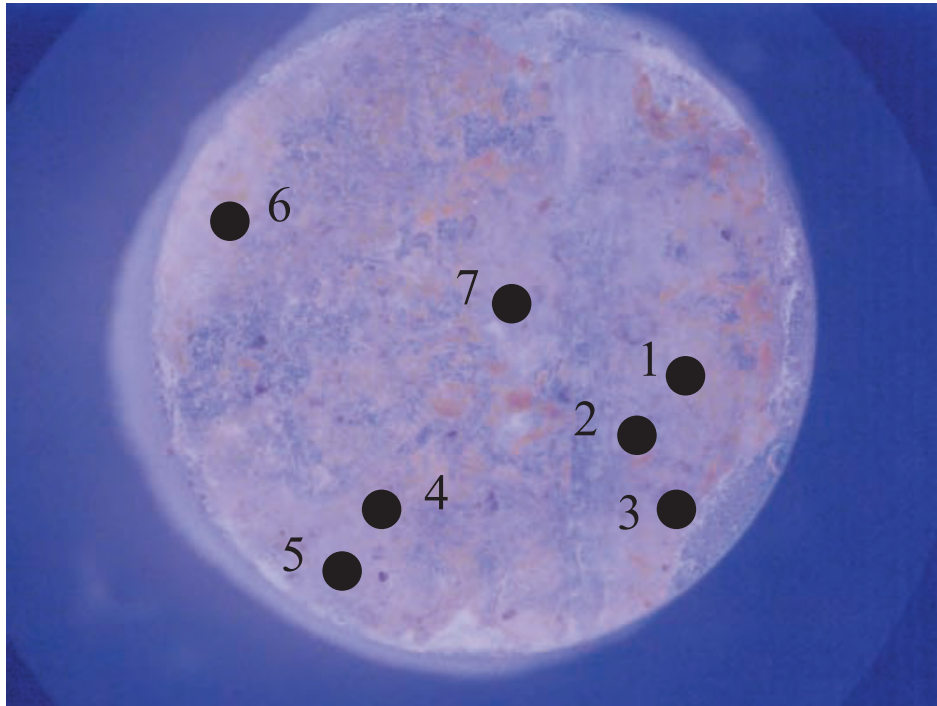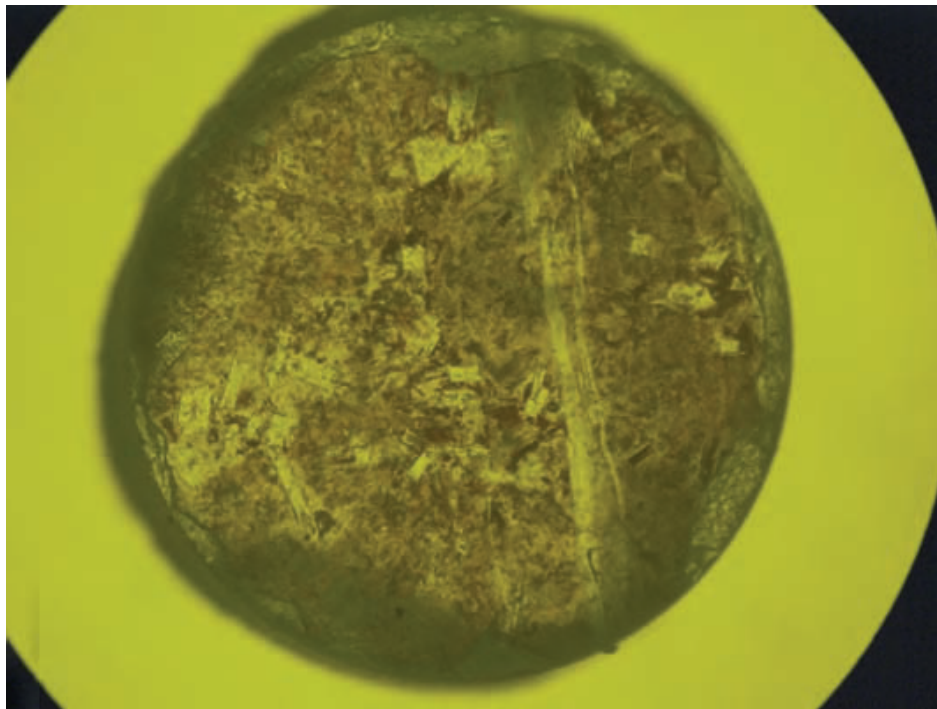

BR46

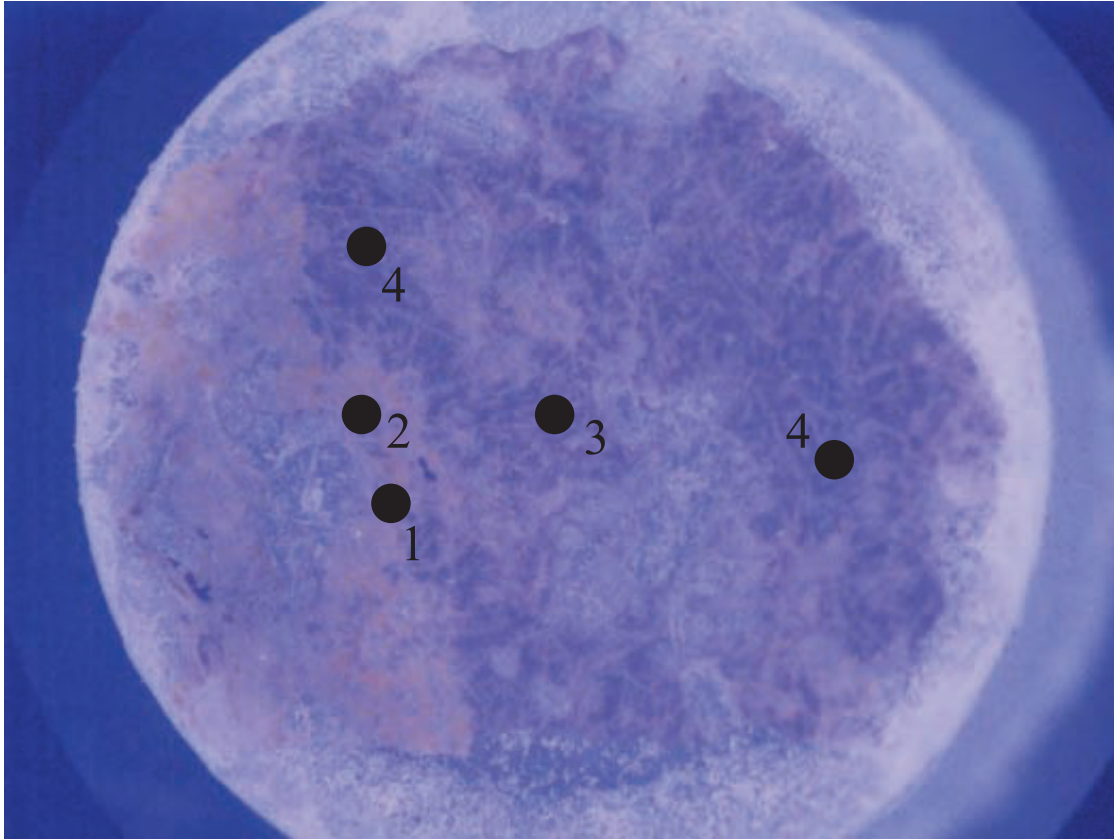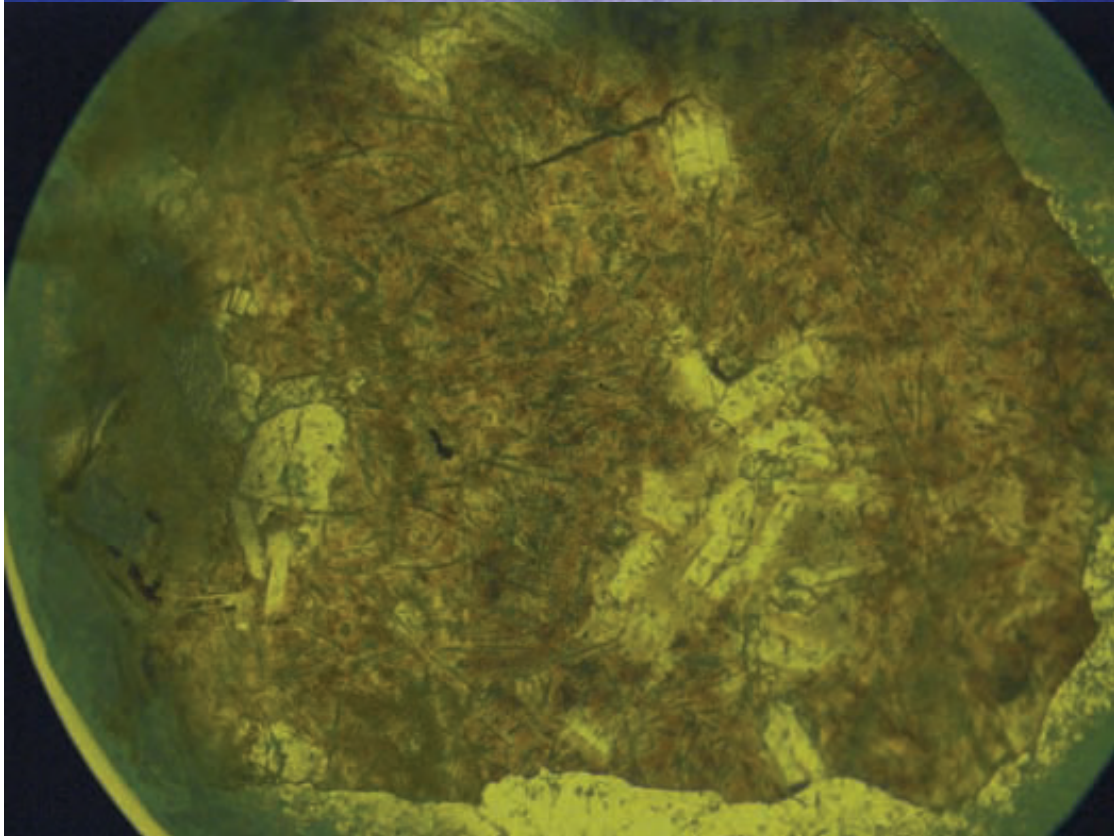

BR119

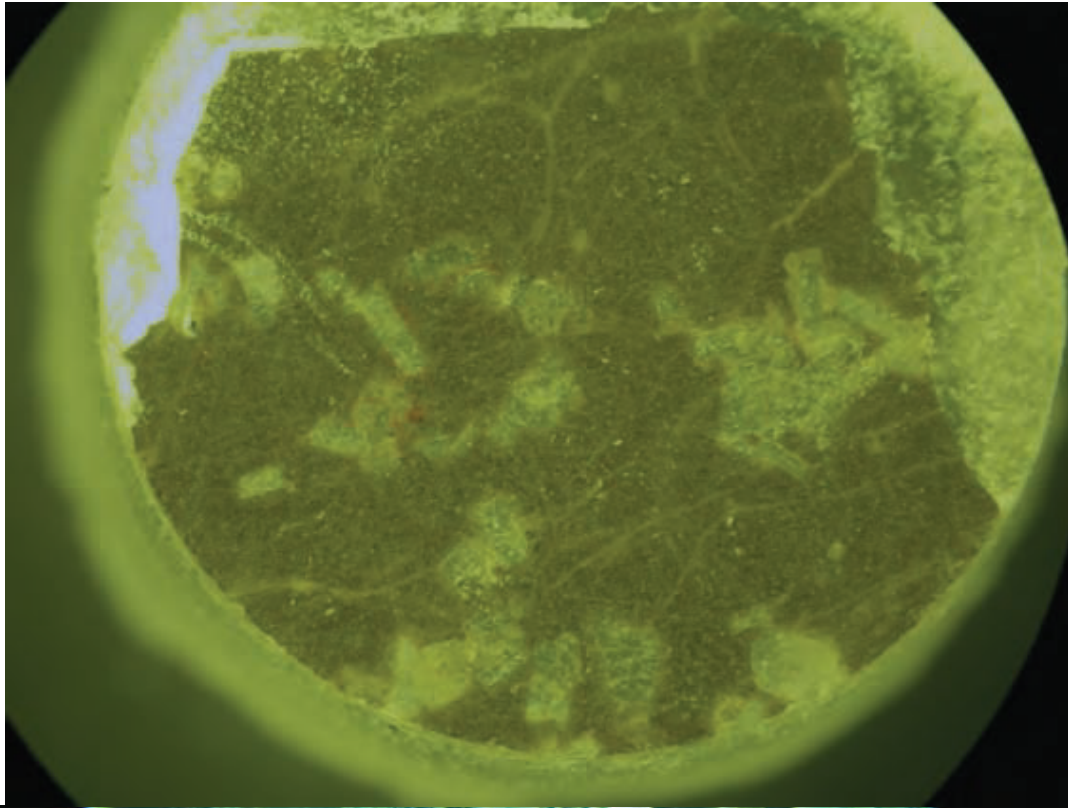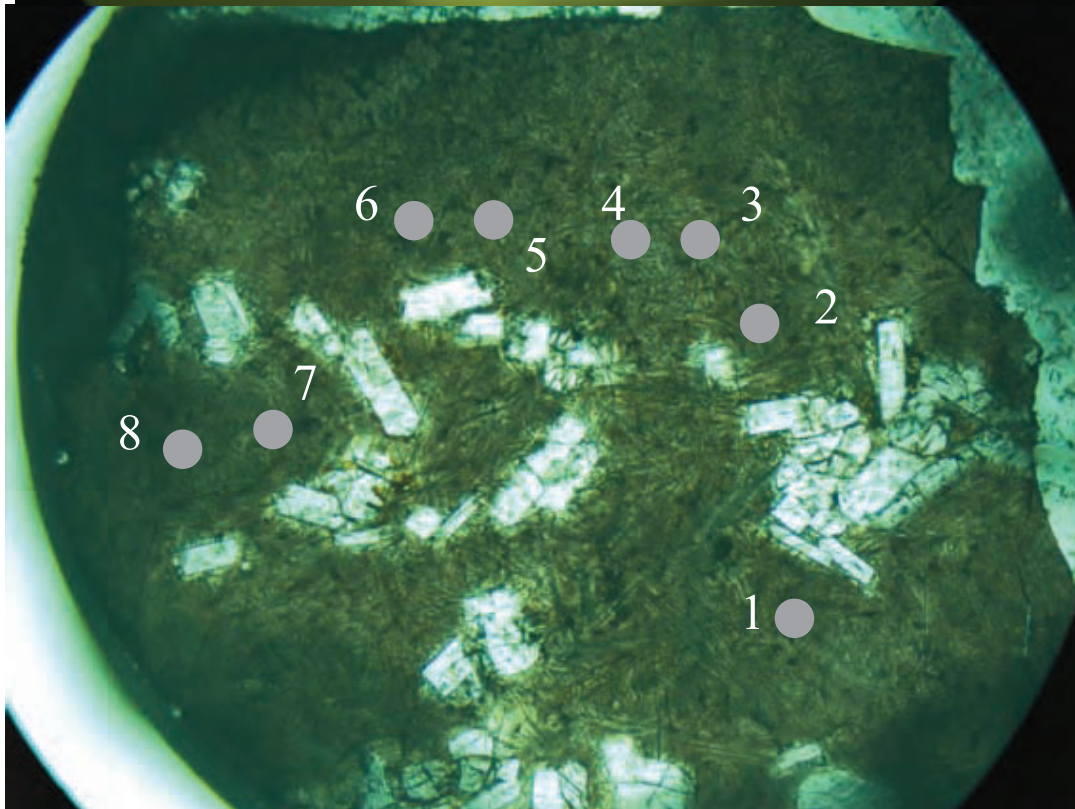

BR120

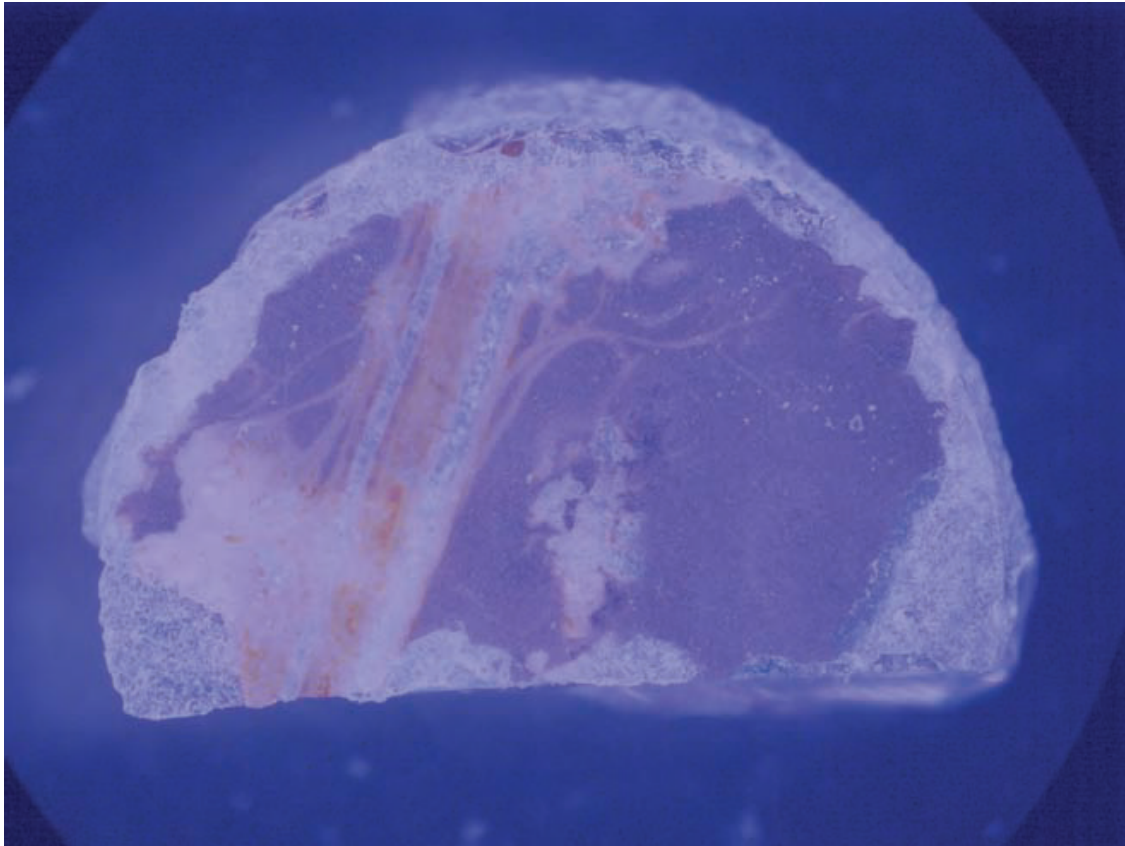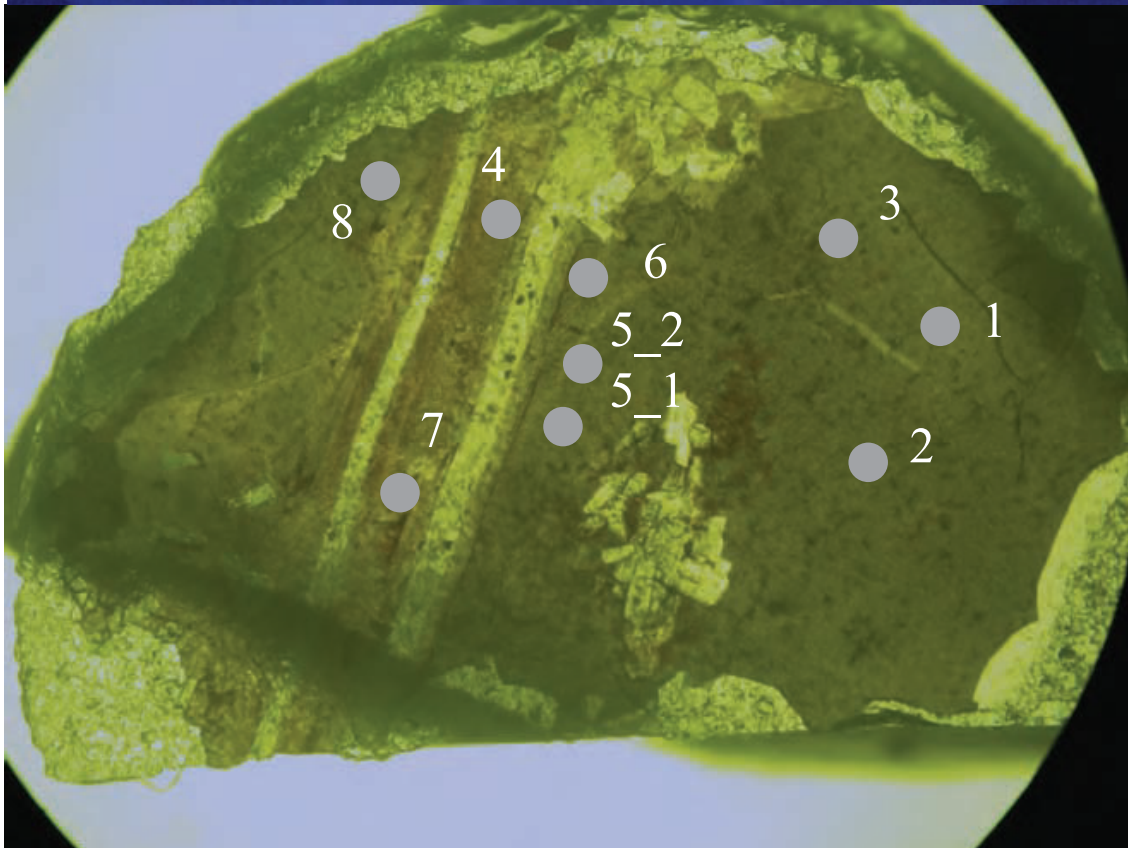

BR122

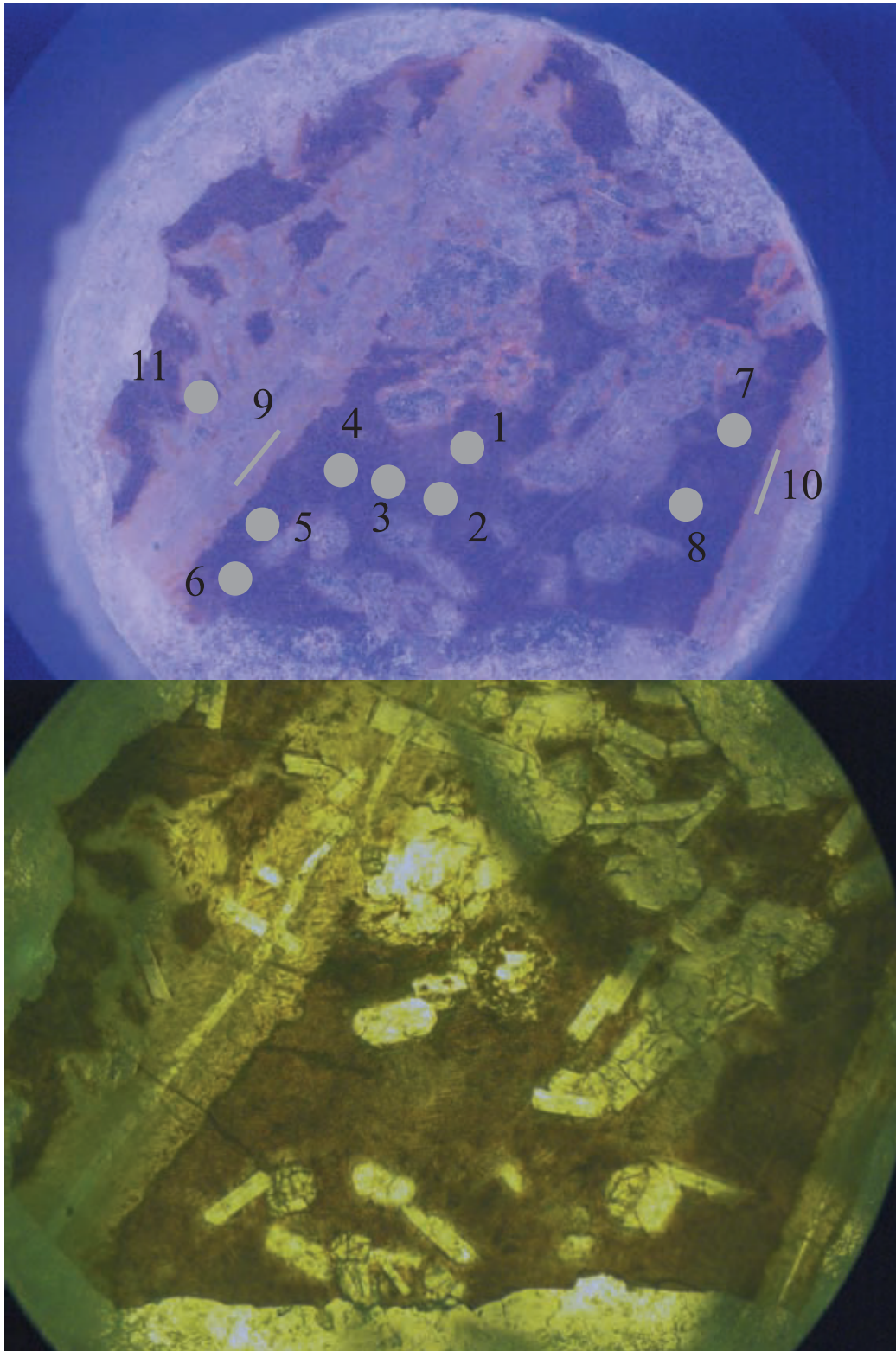

BR124

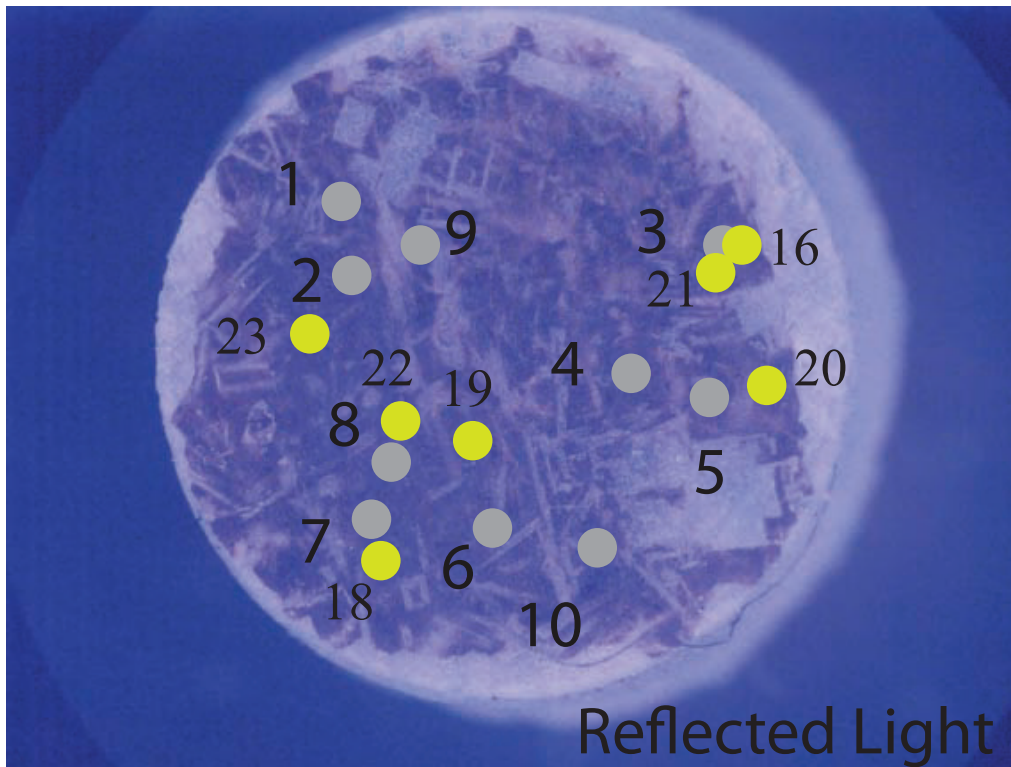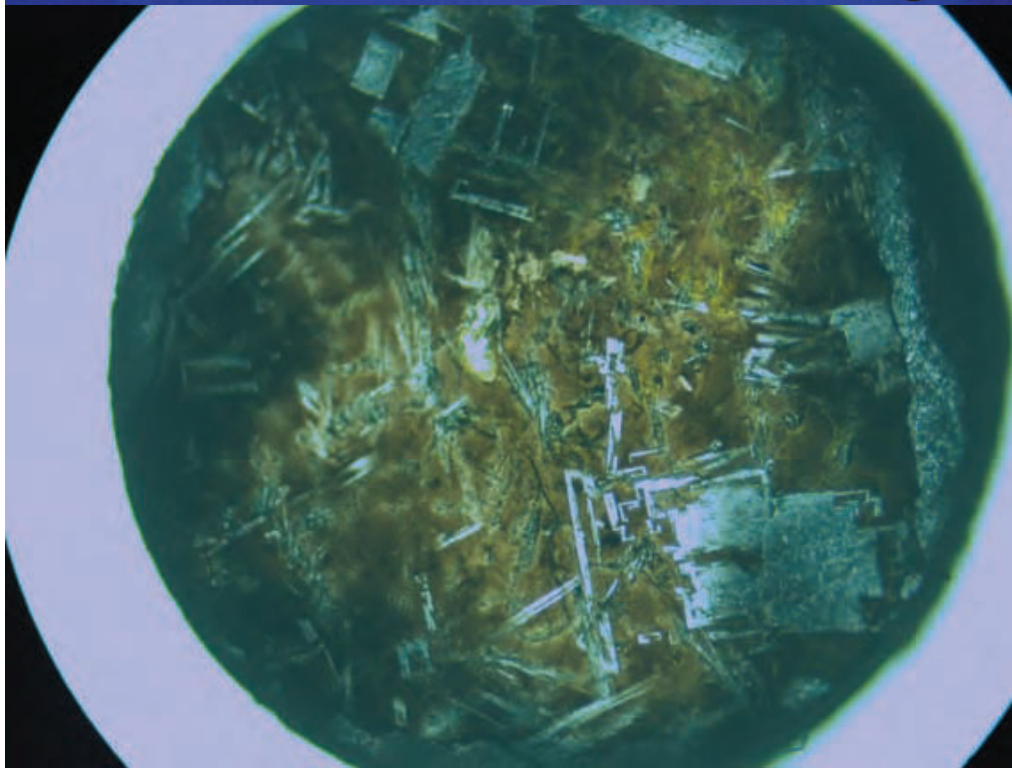

Supplement: Supplementary file 5 — Supplementary Data 3 [file 41467_2022_32736_MOESM5_ESM.pdf]
